# Discovering the Major Antitussive, Expectorant, and Anti-Inflammatory Bioactive Constituents in *Tussilago farfara* L. Based on the Spectrum–Effect Relationship Combined with Chemometrics

**DOI:** 10.3390/molecules25030620

**Published:** 2020-01-31

**Authors:** Liu Yang, Hai Jiang, Song Wang, Ajiao Hou, Wenjing Man, Jiaxu Zhang, Xinyue Guo, Bingyou Yang, Haixue Kuang, Qiuhong Wang

**Affiliations:** 1 Key Laboratory of Chinese Materia Medica, Heilongjiang University of Chinese Medicine, Ministry of Education, Harbin 150040, China; jianghai_777@126.com (H.J.); songwang_yy@163.com (S.W.); Hou_Ajiao@163.com (A.H.); mm1532326@163.com (W.M.); zhang15765312931@163.com (J.Z.); m17645028606@163.com (X.G.); ybywater@163.com (B.Y.); 2 School of Traditional Chinese Medicine, Guangdong Pharmaceutical University, Guangzhou 528458, China

**Keywords:** spectrum–effect relationship, main active compositions, raw and processed Farfarae Flos, chemometrics, grey relational analysis, partial least squares regression

## Abstract

Farfarae Flos (FF) is the dried flower bud of *Tussilago farfara* L, which has antitussive, expectorant, and anti-inflammatory effects. However, little research on the main active composition of FF has been reported. The purpose of this study is to find the main active compounds responsible for the three pharmacological effects (i.e., antitussive, expectorant, and anti-inflammatory effects) of Farfarae Flos, based on the spectrum–effect relationship combined with chemometrics. First, this study uses the UPLC-QDA method to establish the chromatography fingerprint of Farfarae Flos, which is combined with chemometrics to analyze 18 batches of samples. Then, we study the antitussive, expectorant, and anti-inflammatory effects of Farfarae Flos. Finally, the spectrum–effect relationship between the fingerprint and the three pharmacological effects are studied by grey correlation analysis and partial least squares regression. The results show that four, four, and three main active constituents were found for the antitussive, expectorant, and anti-inflammatory pharmacological effects, respectively. In conclusion, we found the main active compounds corresponding to the main pharmacodynamic effects of Farfarae Flos. To our knowledge, this is the first time that spectrum–effect relationships in FF have been established using both raw and processed samples, which provides an experimental basis for further studies on the pharmacodynamic material basis of Farfarae Flos, as well as providing reference for the comprehensive evaluation of Farfarae Flos quality and the development of substitute resources.

## 1. Introduction

The history of traditional Chinese medicine (TCM) can be traced back thousands of years, when people discovered that the roots or bark of some plants were able to treat certain diseases [1]. Compared with Western medicine, TCM has the advantages of low cost, low side effects, and flexible medication. However, the compounds in TCMs are complex and the active compositions are not exactly known [2,3,4]. It is difficult to evaluate the quality of a traditional Chinese medicine comprehensively, as only one or two main active compositions are typically specified in the literature. Fortunately, the emergence of chromatographic fingerprint technology has solved this problem, to some extent.

A chromatographic fingerprint can reveal the chemical characteristics of drugs and is an effective method for evaluating the similarity and quality of TCMs [5,6,7,8]. However, this technique has difficulties in finding the main active constituents of TCMs in response to certain pharmacological effects. Therefore, in order to solve this problem, scholars have found that mathematical statistical methods can be used to establish spectrum–effect relationships and find the effective constituents corresponding to certain pharmacodynamic effects in TCMs [9,10,11,12,13]. For example, by using the spectrum–effect relationship, Liu et al. found that the 11 components of Fangji Huangqi Tang are the main constituents in anti-adriamycin nephropathy [14] and Wang et al. found that the three constituents of Collagen Peptides were the main antioxidants [15]. In this paper, we will explore the main active antitussive, expectorant, and anti-inflammatory constituents in Farfarae Flos, based on the spectrum–effect relationship.

Farfarae Flos (FF) is the dried flower bud of *Tussilago Farfara* L., which grows wildly in China; locals call it “Kuandonghua”. In China, it is distributed in the Hubei, Hunan, Jiangxi, Guizhou, Yunnan, and Gansu provinces. Studies on its phytochemistry and pharmacology have shown that the main active constituents contributing to the antitussive, expectorant, and anti-inflammatory effects of FF are phenolic acids and flavonoids [16,17,18,19]. Many scholars have found multiple compounds in FF [20], but a method is still needed to screen out the main active compounds from a large number of compounds. In addition, we found that, when treating diseases, people usually use honey-processed FF. Processing can change the chemical composition of TCMs, but it is still difficult to identify which are the main active constituents in the chemical composition which change after processing. Therefore, it is necessary to use both raw and processed samples to establish the spectrum–effect relationships and find the main active constituents of FF.

In this paper, the UPLC-QDA method was used to establish the chromatography fingerprint of raw and processed FF and to carry out a similarity analysis (SA) among these samples [21]. Then, principal component analysis (PCA) and hierarchical cluster analysis (HCA) were performed to classify 18 batches of FF, following which 10 representative batches of FF (six batches of raw FF and four batches of processed FF) were screened to study their antitussive, expectorant, and anti-inflammatory effects [22]. Finally, the spectrum–effect relationships of raw and processed FF were established based on grey relational analysis (GRA) and partial least squares regression analysis (PLSR). In this paper, the spectrum–effect relationship was established by using both raw and processed FF for the first time, in order to form a more effective research model of the spectrum–effect relationships of a traditional Chinese medicine.

## 2. Results and Discussion

### 2.1. Results of UPLC Fingerprints

#### 2.1.1. Optimization of UPLC Chromatographic Conditions

In this study, we optimized the Thermo Hypersil GOLD column (100 mm × 2.1 mm, 1.9 µm) (Thermo Scientific TM, Waltham, MA, USA) and the Waters Acquity UHPLC HSS T3 column (50 mm × 2.1 mm, 1.8 µm) (Waters, Milford, MA, USA). The results showed that the Waters Acquity UHPLC HSS T3 column (50 mm × 2.1 mm, 1.8 µm) had a better separation effect and more chromatography peaks. Furthermore, we tested different mobile phase conditions, such as methanol/water and acetonitrile/water. The combination of acetonitrile and 3% aqueous formic acid (*v/v*) was selected as the best mobile phase for separation in this experiment. Gradient optimization was carried out for the liquid phase method, in order to obtain more peaks and good separation in the fingerprint.

#### 2.1.2. UPLC Fingerprints of FF Samples

**Figure 1:**
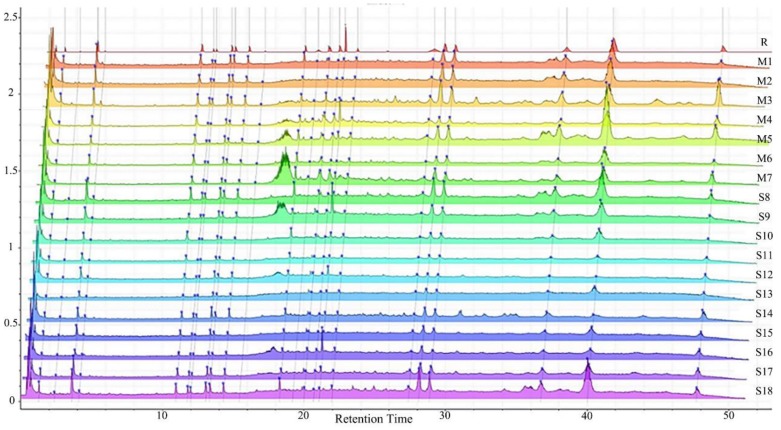
Chromatography fingerprint of 18 batches of samples.

UPLC fingerprints of 18 batches of FF samples were obtained under the optimized condition. According to the length of the retention time, 14 compounds were labeled (as P1–P14). Their compound, retention times, Precursor (*m/z*), and chemical structures are shown in Table 1. After comparison with the standard, they were determined as 3-CQA, P2, Rutin, P4, 3,5-CQA, 3,4-CQA, 4,5-CQA, Kaempferol, P9, P10, P11, P12, P13, and P14. The results of the fingerprint are shown in Figure 1.

The results of the method validation showed that the RSD of RRT and RPA were less than 5% and 6%, respectively, indicating that the precision, repeatability, and stability were good. The chromatography fingerprint method is accurate, effective, and suitable for sample analysis. The results of the RSD are shown in Table 2.

The result of the fingerprint indicates that all samples were genuine from a chemical level. The FF had several common peaks, which could effectively identify the authenticity of the sample. Through the area and proportion of the main characteristic peaks of the fingerprint, the quality of samples can be effectively controlled and relatively stable.

#### 2.1.3. SA of Fingerprints

The SA results are shown in Table 3. The SA of FF samples from different batches were evaluated, and the results show that the SA of samples from S11 (hubei) and S16 (hebei) were less than 0.80. Other batches of samples had good similarities. However, even in the same TCM, the content of different compounds was not the same; the region may be the reason for these differences. Therefore, it is necessary to carry out quality control and investigate the pharmacodynamics of FF.

#### 2.1.4. Results of Hierarchical Cluster Analysis (HCA)

**Figure 2:**
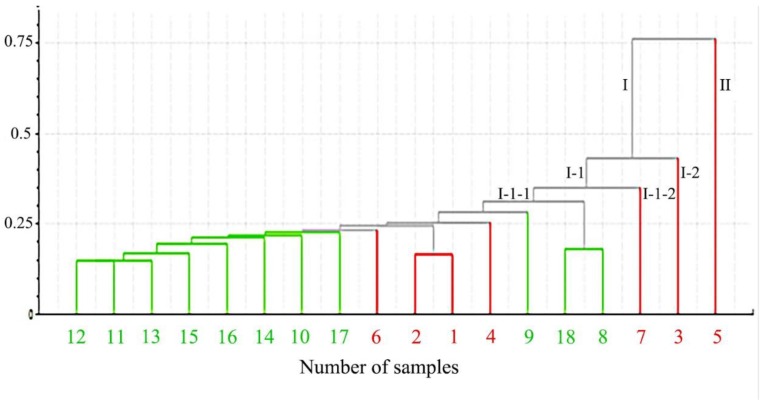
HCA results for 18 batches of samples.

The HCA results are shown in Figure 2. The green lines are raw samples and the red lines are processed samples. HCA analysis of the 18 batches of samples shows that the raw samples S10, S11, S12, S13, S14, S15, S16, and S17 are clearly divided into a cluster, while the processed samples M1, M2, M3, M4, M5, M6, and M7 are clearly classified into another cluster. However, the raw samples S8, S9, and S18, which were purchased from the Anhui province, belonged to the cluster of processed samples. Therefore, we infer that the samples from the Anhui province may have been processed samples which were mistakenly sold as raw samples. This indicates that HCA is a fast and accurate method for identifying raw and processed samples. In the following pharmacological experiments, we selected samples from the cluster of raw samples (S10, S11, S12, S13, S14, S15, S16, and S17), the cluster of processed samples (M1, M2, M3, M4, M5, M6, and M7), and the Anhui samples (S8, S9, and S18).

#### 2.1.5. Results of PCA

**Figure 3:**
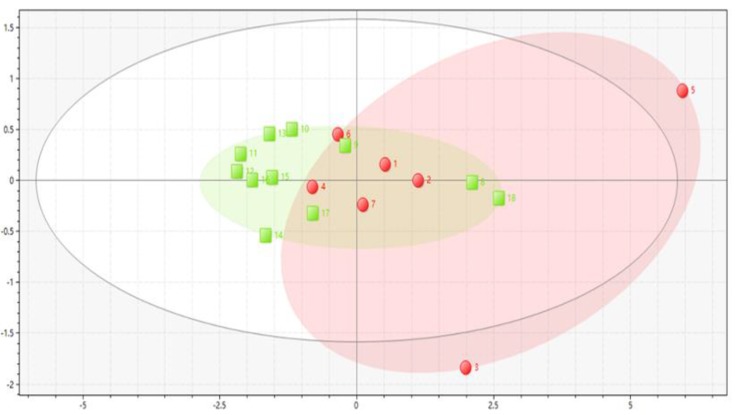
Principal component analysis (PCA) results for the 18 batches of samples.

The PCA results are shown in Figure 3, where the green dots are raw samples and the red dots are processed samples. PCA results show that the Anhui raw samples (S8, S9, and S18) were more similar to the processed samples, which is consistent with our previous inference. The scores of M3 and M5 in the processed samples differed greatly from those of the other processed samples (M1, M2, M4, M6 and M7). We infer that this was because the processing technology of each batch of processed samples is different, which leads to differences in the content of compounds in the samples. Therefore, it is necessary for us to conduct processing technology experiments in the future and study the best processing program for FF. Therefore, it is necessary to study M3 and M5 in the following pharmacological experiments.

#### 2.1.6. Results of Screening Differences Samples

According to the results of the fingerprint, SA, HCA, and PCA, 10 batches of representative samples were screened for the following pharmacological experiments; the 10 batches were re-numbered, which is shown in Table 4. We selected the peak area of 14 common peaks from 10 batches of samples to prepare for the establishment of the spectrum–effect relationship.

### 2.2. Results of Pharmacological Experiments

#### 2.2.1. Results of Antitussive Experiments

**Figure 4:**
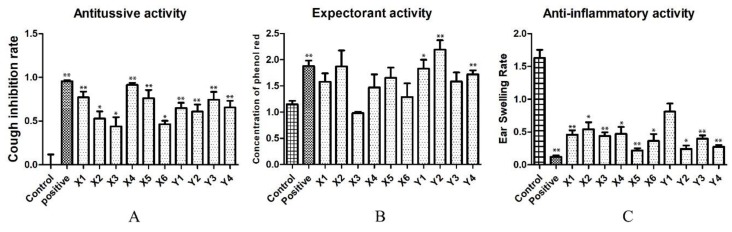
(**A**) Results of antitussive activity test, (**B**) results of expectorant activity test, and (**C**) results of anti-inflammatory activity test (*t*-test; * *p* ≤ 0.05, ** *p* ≤ 0.01). The positive drug groups are pentoxyverine, ammonium chloride, and dexamethasone, respectively. X1–X6: raw sample group, Y1–Y4: processed sample group.

The number of coughs in two minutes after mice were stimulated by ammonia is an important index to evaluate the efficacy of antitussive drugs. Compared with the control group, the number of coughs in the administration group was significantly reduced, indicating that the drug had an antitussive effect. However, the cough inhibition rate in each group was significantly different, which was speculated to have been caused by the difference in the content of various compounds in the drug. The results are shown in Figure 4A.

#### 2.2.2. Evaluation of Expectorant Experiments

Phenol red is expelled from the body along with the respiration of mice. Therefore, the determination of phenol red content in the trachea of mice can be used to determine the expectorant effect of a drug, where the amount of phenol red content in the trachea is positively correlated with the expectorant effect. The results are shown in Figure 4B.

The results show that the processed samples had a good expectorant effect. However, compared with the processed product, there was a huge difference in the expectorant function of the raw drug, which further confirmed the positive effect of processing on FF. We speculate that this may be caused by an increase in the main active constituents in the processed samples.

#### 2.2.3. Evaluation of Anti-Inflammatory Experiments

In this experiment, ear swelling rate was used as the index to evaluate the anti-inflammatory effect, where ear swelling rate = (weight of right ear - weight of left ear)/weight of left ear. The drugs demonstrated a good anti-inflammatory effect, but there were differences in the degree of anti-inflammatory activity. The results are shown in Figure 4C.

### 2.3. Analysis of Spectrum–Effect Relationships

#### 2.3.1. Results of Grey Relational Analysis

Using the 14 compounds as factors, the correlation coefficients between factors and pharmacological effects were calculated. The experimental results of GRA are shown in Table 5. The sequence obtained by GRA was P5 > P8 > P12 > P7 > P14 > P10 > P9 > P3 > P4, which indicates that P5 had the strongest antitussive effect, while the impacts of P2 and P1 were minimal. In addition, the effects of P11, P13, and P6 should not be ignored.

In expectorant effect, the result of GRA was P14 > P9 > P12 > P10 > P11 > P4 > P3 > P2 > P7 and, thus, P14 had the strongest expectorant effect. The GRA results of P13, P6, P1, P5, and P8 were not obvious.

The inhibitory effects on inflammation of the 14 common peaks were in the following order: P14 > P9 > P2 > P13 > P10 > P11 > P12 > P3 > P4 > P1. The results indicate that P14 had the strongest anti-inflammatory effect, while the impact of P7 was minimal; furthermore, the common peaks of P8, P6, and P5 showed a more general effect.

Therefore, we believe that, in antitussive effect, the main active components of FF are P5, P8, P12, P7, and P14. In expectorant effect, the main active components of FF are P14, P9, P12, and P10. In anti-inflammatory effect, the main active components of FF are P14, P9, P2, P13, and P10. However, the correlation coefficients were generally low, which we inferred to potentially be linked to the relative concentrations. However, we had already administered a large dose of the drug, so we believe it may be due to the poor absorption of the active components of FF in mice, so it is necessary to study whether there are drugs or other ways to promote the absorption of FF in the future.

#### 2.3.2. Results of Partial Least Squares Regression Analysis

**Figure 5:**
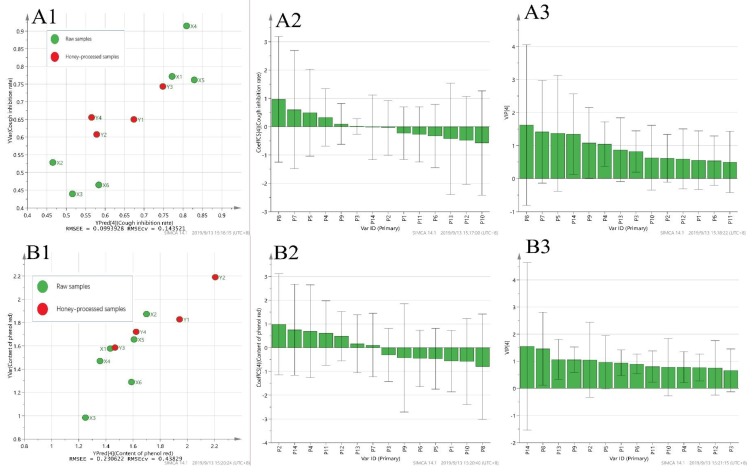

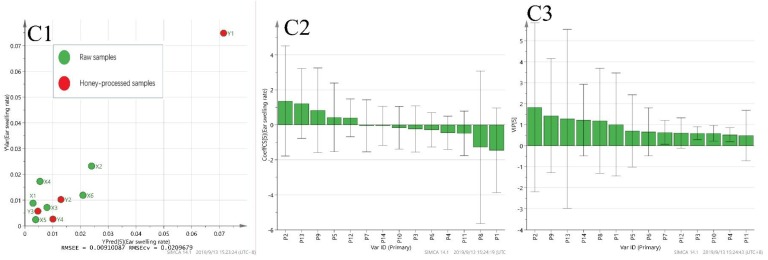
The analysis of three pharmacological experiments by PLSR: PLS linear regression (**A1**, **B1**, **C1**), regression coefficients (**A2**, **B2**, **C2**), and the VIP (**A3**, **B3**, **C3**) of the 14 compounds analyzed.

PLSR analysis is a multiple regression model, combining multiple data fusion and principal component analysis. The regression equation is obtained using the Simca-P (Simca-P14.1, Umetrics, Umeå, Sweden) software, and the principal components are extracted to make the regression model linear. The degree of correlation between X (concentrations of the 14 compounds) and Y (drug efficacy) was determined by using the regression coefficient and the VIP value together as an index. When four main components were extracted, the models corresponding to antitussive and expectorant effects showed a linear relationship. When five main components were extracted, the model corresponding to the anti-inflammatory effect was linear. The results obtained are shown in Figure 5. 

The PLSR results showed that the main active constituents with antitussive effect were P8, P7, P5, P4, and P9. The main active constituents with expectorant effect were P2, P14, P4, and P11. The main active constituents with anti-inflammatory effect were P2, P13, P9, P5, and P12. These results were not completely consistent with the results of GRA, so it is necessary to use a variety of mathematical statistical methods to comprehensively evaluate and study the spectrum–effect of FF, in order to improve the accuracy of the results.

### 2.4. Discussion

This paper has proved that both raw and processed FF have antitussive and anti-inflammatory effects. And FF has expectorant effect after processing. Then the spectrum–effect relationship of the three pharmacological effects were established by GRA and PLSR. In this study, a variety of ingredients with the highest contribution to the three pharmacological effects were identified. To our knowledge, this is the first time to establish the spectrum–effect relationship using both raw and processed FF.

As a complex system with multiple components and multiple targets, synergistic or antagonistic interactions among the different components of TCM should be considered. Therefore, it is more scientific and reasonable for the study of the spectrum–effect relationship. However, the screening of active ingredients by the spectrum–effect relationship still needs to be verified by subsequent experiments.

Previous studies have shown that the four caffeoylquinic acids in FF have antitussive, expectorant and anti-inflammatory effects [17]. In this study, we found not only four phenolic acids with these pharmacological effects, but also some other compounds with higher contribution. In the following study, it will be necessary to study the mechanism of these compounds at the molecular level.

In this study, the spectral–effect relationship can screen the active compounds of FF comprehensively and maintain consistency between medical and biological effects. Laid the foundation for the development of new drugs and discovery of lead compounds.

In the future, we will carry out qualitative and quantitative research on unknown compounds with high contributions. We will then conduct validation experiments in vivo and in vitro. Finally, the mechanism of these compounds at the molecular level was studied. We expect this experiment will provide a precedent for other TCM and found more compounds that respond to certain pharmacological effects.

## 3. Materials and Methods

### 3.1. Instruments

Acetonitrile (UPLC grade) was obtained from Fisher Scientific International (USA). A microplate reader was obtained from BioTek (Winooski, VT, USA).

### 3.2. Materials and Reagents

In the medicinal materials market, 18 batches of FF samples (7 batches of honey samples and 11 batches of raw samples) from various regions of China were purchased and appraised by Professor Su Lianjie of the Heilongjiang University of Traditional Chinese Medicine. They were all the Chinese herbal medicine Farfarae Flos. Table 6 shows the originating regions of the 18 batches of samples.

Six standards (3,5-CQA, 3,4-CQA, 4,5-CQA, 3-CQA, rutin, and kaempferol) were purchased from the Shanghai Nature Standard Technology Co., Ltd. (Shanghai, China) and the purity was chromatographic grade. Water was purchased from Hangzhou Wahaha Company. Other reagents were all above 98% purity.

### 3.3. Animals

ICR mice (20–22 g) were purchased from the GLP of the Heilongjiang University of Chinese Medicine (Harbin, China). All mice were given free access to food and water under a standard light–dark cycle. The indoor temperature was 25 ± 2 °C and relative humidity was 50% ± 10%. The animal study was performed according to the international rules considering animal experiments and the internationally accepted ethical principles for laboratory animal use and care. Animal welfare and experimental procedures were carried out in accordance with the ethical regulations of Heilongjiang University of Chinese Medicine (License number: SCXK (Hei) 2013-004.).

### 3.4. UPLC-QDA Fingerprints

#### 3.4.1. Establishment of UPLC Conditions

The Waters ACQUITY UPLC system (Waters, Milford, MA, USA) was used for fingerprint analysis, which was equipped with a vacuum degasser, quaternary pump, sample manager, and single quadrupole mass spectrometry (QDA) detector (Waters, Milford, MA, USA). The chromatogram was analyzed using a Waters ACQUITY T3 column (50 mm × 2.1 mm, 1.8 μm) at 40 °C. The mobile phase consisted of acetonitrile (A) and water containing 0.3% formic acid (B). The optimized gradient condition was: 0–14 min: 5%–22% A, 14–15 min: 22%–30% A, 15–17 min: 30–40% A, 17–20 min: 40–50% A, 20–22 min: 50–55% A, 22–31 min: 55%–60% A, 31–43 min: 60%–64% A, 43–49 min: 64%–80% A, 49–51 min: 80% A. The flow rate was set to 0.3 mL/min and volume injected was set as 2 µL.

#### 3.4.2. Preparation of Sample Solutions

A 5 g FF sample was added into a 50 mL centrifuge tube, to which 50 mL of 85% methanol water was added. According to the method in the literature, ultrasonic extraction was performed for 60 min (60 kHz, 500 W). After centrifugation, the supernatant was filtered by a 2.2 µm filter membrane and stored in a refrigerator at 4 °C for further analysis. All samples were treated using the above method.

#### 3.4.3. Preparation of Standard Solutions

The six standards (3,5-CQA, 3,4-CQA, 4,5-CQA, 3-CQA, rutin, and kaempferol) were accurately weighed to prepare the original solution with a concentration of 1.0 g/mL. These were stored in a refrigerator at 4 °C for further analysis.

#### 3.4.4. Method Validation of Fingerprint Analysis

ChemPatternTM software (version 2017, Chemmind Technologies. Co., Ltd., Beijing, CHN) was used to obtain the fingerprint, which was then verified for accuracy, repeatability, and stability. Both relative retention time (RRT) and relative peak area (RPA) are available in the software. Precision: six consecutive analyses were performed using the same method. Repeatability: the sample was tested 6 times. Stability: the samples were measured at 0, 2, 4, 6, 8, 12, and 24 h.

#### 3.4.5. Similarity Analysis of UPLC Fingerprints

To establish the representative chromatographic fingerprint, a total of 18 *Tussilago farfara* L. samples (7 batches of honey-processed *Tussilago farfara* L. (M1–M7) and 11 batches of raw *Tussilago farfara* L. (S8–S18)) were analyzed by the above method. The chromatography fingerprints of FF were established using the ChemPatternTM software (version 2017, Chemmind Technologies. Co., Ltd., Beijing, CHN).

#### 3.4.6. PCA

Principal component analysis (PCA) is a statistical analysis method which reduces multiple indicators into a few comprehensive indicators [23]. In the study of multiple indicators (variables), it is often used because the number of variables is too large, and there are certain correlations between them, such that the observed data overlaps, to some extent. When there are many variables, it is more troublesome to study the distribution of samples in high-dimensional space. PCA adopts a dimension reduction method to find several comprehensive factors to represent the original variables, such that these comprehensive factors reflect the information quantity of the original variables as much as possible and are not related to each other, thus simplifying the analysis. In this paper, PCA of different batches of FF samples was carried out using the ChemPatternTM software (version 2017, Chemmind Technologies. Co., Ltd., Beijing, CHN). 

#### 3.4.7. HCA

The function of HCA is to establish a classification method that classifies a batch of samples or variables according to their degree of intimacy in nature. The ChemPatternTM software (Chemmind Technologies. Co., Ltd.) was used to establish the HCA model.

### 3.5. Experiments of Pharmacodynamic Effects

In this paper, 10 batches of representative drugs (4 batches of honey-processed FF (Y1–Y4) and 6 batches of raw FF (X1–X6)) were screened by SA, PCA, and HCA, where antitussive, expectorant, and anti-inflammatory pharmacodynamic effects were investigated.

#### 3.5.1. Preparation of Gavage

The FF was placed in eight volumes of ethanol and heated for 1.5 h. The liquid was taken out and the process was repeated three times. All liquids were combined and concentrated to an appropriate volume using a rotary evaporator, and the result was stored in a refrigerator at 4 °C for further analysis.

#### 3.5.2. Evaluation of Antitussive Activity

We studied the effect of FF on cough in mice caused by ammonia liquor. A medical cotton ball was moistened with 1.5 mL of ammonia liquor, placed in a 1000 mL beaker with the mice, covered with a glass plate, and the cough latency of the mice was recorded. One minute later, the mice were transferred to a clean 1000 mL volume beaker and the number of coughs in the mice over 2 min was recorded. Mice with an incubation period of less than 1 min and a cough frequency of more than 3 times in 1 min were selected as experimental animals. After 24 h of recovery, 48 mice were randomly divided into 12 groups and given the test drugs (91 g/kg) for 5 days, using pentoxyverine (1.75 mg/kg) as the positive control. One hour after the last administration, the experiment was carried out by the above method. The same skilled operator was used for the experiment and recording.

#### 3.5.3. Evaluation of Expectorant Activity

Using the characteristics of partial excretion from the airway after intraperitoneal injection of phenol red by mice, the amount of phenol red excretion in the airway of mice was used to determine the effect of the test drug on airway permeability and secretion volume. To understand the effect of FF on mice, a total of 48 mice were randomly divided into 12 groups and administered continuously for 5 days. The positive drug used ammonium chloride (1 g/kg). The mice were starved for 16 h before the experiment and were only given water. The test drug was intragastrically administered 30 min before the injection of phenol red. Then, the mice were intraperitoneally injected with 0.2%/10 g of 1% phenol red physiological saline solution. The neck was sacrificed 30 min after injection. The blood in the mice was left to coagulate, then the trachea was removed and 1.5 mL of 2% sodium bicarbonate solution was immediately added. The sample was sonicated for 15 min and centrifuged at 2500 r for 5 min. Finally, the supernatant was taken and the absorbance at 545 nm was measured using a microplate reader.

#### 3.5.4. Evaluation of Anti-Inflammatory Activity

The anti-inflammatory activity of FF was determined by the degree of swelling of the mouse ear after application of xylene. Forty-eight mice were randomly divided into 12 groups and administered continuously for 5 days. The positive drug was dexamethasone (10.5 mg/kg). Thirty minutes after the last administration of the test drugs, 0.02 mL of xylene was uniformly applied to the front and rear surfaces of the right ear, and an equal volume of water was applied to the left ear. After 30 min, the neck was sacrificed, and a disc was weighed with a 6 mm puncher at the same position of both ears. The weight of each right ear disc minus the weight of the left ear disc was used to calculate the degree of swelling.

### 3.6. The Spectrum-Effect Relationship

The chemical fingerprint addresses the problem of complex composition in TCM, to a certain extent, but the fingerprint cannot determine all the active ingredients in TCM and cannot confirm the effective ingredients with a certain efficacy. Therefore, the use of fingerprints to evaluate the quality of traditional Chinese medicines is obviously not enough. However, with the development of fingerprints, the fingerprints can be linked to the therapeutic effects by chemometric methods; this concept is called the “spectrum–effect relationship” method. This paper establishes the spectrum–effect relationships between the fingerprint and the three pharmacodynamic effects. In this paper, grey relational analysis (GRA) and partial least squares regression analysis (PLSR) are used to establish the spectrum–effect relationship between fingerprint and three pharmacodynamics.

#### 3.6.1. Grey Relational Analysis (GRA)

Grey relational analysis (GRA) is a quantitative description and comparison of the evolution of a system. It can solve for the relationships in complex multivariate and multi-factor situations. In this paper, the data processing system (version 7.05., Hangzhou Ruifeng information technology co. LTD, Hangzhou, China) software was used to study the correlation between the three effects and 14 common peaks in the FF samples, and to find the main active constituents corresponding to the pharmacological effects [24].

#### 3.6.2. Partial Least Squares Regression (PLSR) Model

Regression, in the modern sense, is a statistical analysis method which studies the dependence of dependent variables on independent variables. The purpose is to estimate or predict the mean of dependent variables by the given values of the independent variables. It can be used for forecasting, time-series modeling, and finding causal relationships between variables. Simply put, regression is used to analyze the relationship between the dependent variable and the independent variable, in order to provide a scientific and reasonable method for analyzing and predicting data. When the amount of data is small (i.e., even smaller than the variable dimension) and the correlation is relatively large, partial least squares regression (PLSR) has very high validity and practicability. In this study, PLSR was used to establish models for 14 common peaks and 3 pharmacodynamics. The Simca-P was applied to establish the PLSR model, thus finding the main active constituents for the three pharmacodynamic effects by looking at the VIP and regression coefficients.

## 4. Conclusions

In this work, UPLC-QDA fingerprints and a series of in vivo antitussive, expectorant, and anti-inflammatory effects were combined to investigate the spectrum–effect relationship of FF. The GRA and PLSR results show that Kaempferol (P8), 4,5-CQA (P7), 3,5-CQA (P5), and P4 were the main active antitussive constituents; P2, P14, P4, and P11 were the main active expectorant constituents; and P2, P13, and P9 were the main active anti-inflammatory constituents. The structures and effects of the substances P2, P14, P4, P11, P13, and P9 require further confirmation: however, we determined their precursors in this paper. These compounds may have potential as drugs for the treatment or prevention of antitussive, expectorant, and anti-inflammatory. The study also demonstrates the value of the spectrum–effect method in FF while also found a more scientific and efficient method to search for active compounds in other TCM. Finally, the exact mechanisms explaining the three pharmacological effects of compounds in FF will be studied in the future.

## Figures and Tables

**Table 1:** Compounds, retention times, Precursor (*m/z*), and chemical structures of the constituents studied.

Peak	Compound	Retention Time (Min)	Precursor (*m*/*z*)	Adduct	Structure
P1	3-CQA (Chlorogenic acid)	3.47	376.98	[M + Na]^+^	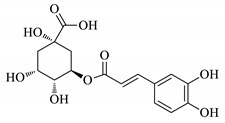
P2	Unknown	10.48	366.35	Unknown	Unknown
P3	Rutin	11.33	633.15	[M + Na]^+^	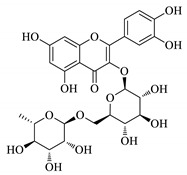
P4	Unknown	11.56	487.28	Unknown	Unknown
P5	3,5-CQA (3,5-Dicaffeoylquinic acid)	12.64	539.21	[M + Na]^+^	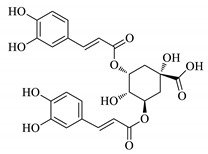
P6	3,4-CQA (3,4-Dicaffeoylquinic acid)	12.98	538.92	[M + Na]^+^	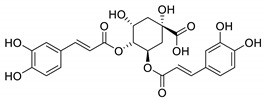
P7	4,5-CQA (4,5-Dicaffeoylquinic acid)	14.04	538.99	[M + Na]^+^	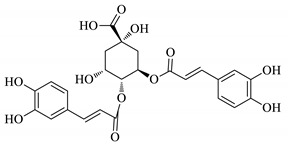
P8	Kaempferol	17.97	286.90	[M + H]^+^	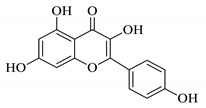
P9	Unknown	27.10	453.21	Unknown	Unknown
P10	Unknown	27.73	413.15	Unknown	Unknown
P11	Unknown	28.58	471.08	Unknown	Unknown
P12	Unknown	36.94	453.14	Unknown	Unknown
P13	Unknown	39.82	513.32	Unknown	Unknown
P14	Unknown	47.60	453.36	Unknown	Unknown

**Table 2:** Results of relative retention times and relative peak areas of precision, repeatability, and stability.

Peak No.	RRT	R.S.D. (%)			RPA	R.S.D. (%)		
		Precision	Repeatability	Stability		Precision	Repeatability	Stability
1	1.000	0.19	1.14	0.92	1.000	3.55	2.85	3.95
2	1.094	0.34	0.28	0.99	0.268	3.85	3.61	2.90
3	1.193	0.33	0.64	0.61	0.918	3.53	3.64	4.23
4	1.216	0.53	0.71	0.55	1.088	4.32	3.01	2.91
5	1.306	0.28	1.62	0.86	0.776	3.74	3.55	4.46
6	1.668	1.13	0.52	0.92	0.825	4.33	3.05	5.02
7	1.752	0.74	0.46	0.91	0.086	4.76	3.92	4.46
8	1.816	0.68	0.89	0.51	0.152	4.28	3.60	4.08
9	2.511	0.44	0.67	0.87	0.535	2.53	2.60	3.73
10	2.593	0.57	1.33	1.03	2.100	2.44	3.36	3.77
11	2.671	0.60	0.73	0.88	2.132	2.04	3.11	3.92
12	3.353	1.05	1.11	0.82	1.744	4.41	4.62	3.92
13	3.654	0.94	0.83	0.69	6.995	3.81	2.76	2.55
14	4.358	0.45	0.37	1.02	0.922	2.48	2.60	4.48

**Table 3:** Similarity analysis of 18 Batches of Farfarae Flos (FF) Samples.

NO.	SA	NO.	SA
M1	0.98	S10	0.91
M2	0.90	S11	0.79
M3	0.92	S12	0.98
M4	0.94	S13	0.92
M5	0.90	S14	0.87
M6	0.93	S15	0.94
M7	0.92	S16	0.74
S8	0.93	S17	0.85
S9	0.92	S18	0.90

**Table 4:** Renumbering of the 10 screened FF sample batches.

Previous Number	Renumber	Previous Number	Renumber
S8	X1	S15	X6
S9	X2	M1	Y1
S10	X3	M3	Y2
S12	X4	M5	Y3
S14	X5	M6	Y4

**Table 5:** Grey relational analysis (GRA) results of antitussive, expectorant, anti-inflammatory experiments.

	Antitussive Effect		Expectorant Effect		Anti-Inflammatory Effect	
Order	Peak	Correlation Coefficient	Peak	Correlation Coefficient	Peak	Correlation Coefficient
1	P5	0.6945	P14	0.7648	P14	0.7307
2	P8	0.6850	P9	0.7481	P9	0.6844
3	P12	0.6821	P12	0.7361	P2	0.6506
4	P7	0.6818	P10	0.7315	P13	0.6495
5	P14	0.6807	P11	0.7267	P10	0.6482
6	P10	0.6645	P4	0.7261	P11	0.6426
7	P9	0.6644	P3	0.7130	P12	0.6393
8	P3	0.6611	P2	0.7044	P3	0.6386
9	P4	0.6557	P7	0.7014	P4	0.6364
10	P11	0.6473	P13	0.6919	P1	0.6337
11	P13	0.6408	P6	0.6868	P8	0.6337
12	P6	0.6380	P1	0.6791	P6	0.6254
13	P2	0.6273	P5	0.6748	P5	0.6228
14	P1	0.6234	P8	0.6550	P7	0.6184

**Table 6:** Origin of the 18 batches of Farfarae Flos samples.

NO.	Origin	NO.	Origin
M1	Yunnan	S10	Gansu
M2	Gansu	S11	Hubei
M3	Sichuan	S12	Hubei
M4	Jiangxi	S13	Hubei
M5	Heilongjiang	S14	Gansu
M6	Hebei	S15	Gansu
M7	Hebei	S16	Hebei
S8	Anhui	S17	Heilongjiang
S9	Anhui	S18	Anhui

## References

[1] Farnsworth N.R. (1988). Screening plants for new medicines. Biodiversity.

[2] Jiang Y., David B., Tu P., Barbin Y. (2010). Recent analytical approaches in quality control of traditional Chinese medicines—A review. Anal. Chim. Acta.

[3] Rooney J.S., McDowell A., Strachan C.J., Gordon K.C. (2015). Evaluation of vibrational spectroscopic methods to identify and quantify multiple adulterants in herbal medicines. Talanta.

[4] Feng G., Sun Y., Liu S., Song F., Pi Z., Liu Z. (2019). Stepwise targeted matching strategy from in vitro to in vivo based on ultra–high performance liquid chromatography tandem mass spectrometry technology to quickly identify and screen pharmacodynamic constituents. Talanta.

[5] Tistaert C., Thierry L., Szandrach A., Dejaegher B., Fan G., Frédérich M., Vander Heyden Y. (2011). Quality control of Citri reticulatae pericarpium: Exploratory analysis and discrimination. Anal. Chim. Acta.

[6] Liang X.M., Jin Y., Wang Y.P., Jin G.W., Fu Q., Xiao Y.S. (2009). Qualitative and quantitative analysis in quality control of traditional Chinese medicines. J. Chromatogr. A.

[7] Xie P.S., Leung A.Y. (2009). Understanding the traditional aspect of Chinese medicine in order to achieve meaningful quality control of Chinese materia medica. J. Chromatogr. A.

[8] Xu C.J., Liang Y.Z., Chau F.T., Heyden Y.V. (2006). Pretreatments of chromatographic fingerprints for quality control of herbal medicines. J. Chromatogr. A.

[9] Xu G.L., Xie M., Yang X.Y., Song Y., Yan C., Yang Y., Zhang X., Liu Z.Z., Tian Y.X., Wang Y. (2014). Spectrum-effect relationships as a systematic approach to traditional chinese medicine research: Current status and future perspectives. Molecules.

[10] Jiang Z., Zhao C., Gong X., Sun X., Li H., Zhao Y., Zhou X. (2018). Quantification and Efficient Discovery of Quality Control Markers for Emilia prenanthoidea DC. by fingerprint-efficacy relationship modelling. J. Pharm. Biomed. Anal..

[11] Wang F., Xiong Z.Y., Li P., Yang H., Gao W., Li H.J. (2017). From chemical consistency to effective consistency in precise quality discrimination of Sophora flower-bud and Sophora flower: Discovering efficacy-associated markers by fingerprint-activity relationship modeling. J. Pharm. Biomed. Anal..

[12] Zhang X.F., Chen J., Yang J.L., Shi Y.P. (2018). UPLC-MS/MS analysis for antioxidant components of Lycii Fructus based on spectrum-effect relationship. Talanta.

[13] Zhu C.S., Lin Z.J., Xiao M.L., Niu H.J., Zhang B. (2016). The spectrum-effect relationship-a rational approach to screening effective compounds, reflecting the internal quality of Chinese herbal medicine. Chin. J. Nat. Med..

[14] Liu X., Wang X., Zhu T., Zhu H., Zhu X., Cai H., Cao G., Xu X., Niu M., Cai B. (2018). Study on spectrum-effect correlation for screening the effective components in Fangji Huangqi Tang basing on ultra-high performance liquid chromatography-mass spectrometry. Phytomedicine.

[15] Wang J., Luo D., Liang M., Zhang T., Yin X., Zhang Y., Yang X., Liu W. (2018). Spectrum-Effect Relationships between High-Performance Liquid Chromatography (HPLC) Fingerprints and the Antioxidant and Anti-Inflammatory Activities of Collagen Peptides. Molecules.

[16] Segheto L., Santos B.C.S., Werneck A.F.L., Vilela F.M.P., de Sousa O.V., Rodarte M.P. (2018). Antioxidant extracts of coffee leaves and its active ingredient 5-caffeoylquinic acid reduce chemically-induced inflammation in mice. Ind. Crop. Prod..

[17] Wu Q.Z., Zhao D.X., Xiang J., Zhang M., Zhang C.F., Xu X.H. (2016). Antitussive, expectorant, and anti-inflammatory activities of four caffeoylquinic acids isolated from Tussilago farfara. Pharm. Biol..

[18] Ge Y., Zhang F., Qin Q., Shang Y., Wan D. (2015). In Vivo Evaluation of the Antiasthmatic, Antitussive, and Expectorant Activities and Chemical Components of Three Elaeagnus Leaves. Evid. Based Complement. Altern. Med..

[19] Cheon H.J., Nam S.H., Kim J.K. (2018). Tussilagone, a major active component in Tussilago farfara, ameliorates inflammatory responses in dextran sulphate sodium-induced murine colitis. Chem. -Biol. Interact..

[20] Yang A., Shang Q., Yang L., Li C., Yuan H.J. (2018). Chemical Constituents of the Flower Buds of Tussilago farfara. II. Chem. Nat. Compd..

[21] Yang L., Jiang H., Xing X., Yan M., Guo X., Man W., Hou A., Yang L. (2019). A Biosensor-Based Quantitative Analysis System of Major Active Ingredients in Lonicera japonica Thunb. Using UPLC-QDa and Chemometric Analysis. Molecules.

[22] Wang L.J., Jiang Z.M., Xiao P.T., Sun J.B., Bi Z.M., Liu E.H. (2019). Identification of anti-inflammatory components in Sinomenii Caulis based on spectrum-effect relationship and chemometric methods. J. Pharm. Biomed. Anal..

[23] Sun L.-L., Wang M., Ren X. (2017). Application progress on chemical pattern recognition in quality control of Chinese materia medica. Chin. Tradit. Herb. Drugs.

[24] Tang Q.-Y., Zhang C.-X. (2013). Data Processing System (DPS) software with experimental design, statistical analysis and data mining developed for use in entomological research. Insect Sci..

